# Short-term changes in soil pore size distribution: Impact of land use

**DOI:** 10.1016/j.still.2020.104597

**Published:** 2020-05

**Authors:** Johannes L. Jensen, Per Schjønning, Christopher W. Watts, Bent T. Christensen, Lars J. Munkholm

**Affiliations:** a Department of Agroecology, Aarhus University, Blichers Allé 20, 8830 Tjele, Denmark; b Department of Sustainable Agriculture Sciences, Rothamsted Research, Harpenden, Hertfordshire AL5 2JQ, United Kingdom

**Keywords:** A, Arable, AG, Arable converted to grass, BF, Bare fallow, BFG, Bare fallow converted to grass, Dex, Double-exponential model, G, Grass, GA, Grass converted to arable, GBF, Grass converted to bare fallow, PAWC_eq_, Plant available water capacity based on identical soil quantities, PSD, Pore size distribution, *V*_2_, Structural void ratio, Land-use change, Pore size distribution, Soil degradation and recovery

## Abstract

Changes in land use affect the pore size distribution (PSD) of the soil, and hence important soil functions such as gas exchange, water availability and plant growth. The objective of this study was to investigate potentially damaging and restorative soil management practices on soil pore structure. We quantified the rate of change in PSD six years after changes in land use taking advantage of the Highfield land-use change experiment at Rothamsted Research. This experiment includes short-term soil degradation and restoration scenarios established simultaneously within long-term contrasting treatments that had reached steady-state equilibrium. The land-use change scenarios comprised conversion to grassland of previously arable or bare fallow soil, and conversion of grassland to arable and bare fallow soils. In the laboratory, we exposed intact soil cores (100 cm^3^) to matric potentials ranging from −10 hPa to -1.5 MPa. Based on equivalent soil mass, the plant available water capacity decreased after conversion from grassland, whereas no change was observed after conversion to grassland. Structural void ratio decreased after termination of grassland and introduction of grassland in bare fallow soil, while no change was seen when changing arable to grassland. Consequently, it was faster to degrade than to restore a complex soil structure. The study illustrates that introducing grassland in degraded soil may result in short-term increase in soil density.

## Highlights

• The rate of change in pore size distribution was quantified.
• It was faster to degrade than to restore a complex soil structure.
• Introducing grassland in degraded soil may induce densification in the short-term.

## 1 Introduction

The soil-water retention curve, i.e. the relationship between the soil water content and soil matric potential, shows the amount of water retained in the soil at a given matric potential. The tube-equivalent pore size at a given matric potential can be approximated by the physics-based capillary rise equation of Young-Laplace. Management effects on pore size distribution (PSD) have been reported in several papers (e.g., Dexter and Richard, 2009; Reynolds et al., 2009). The PSD of the soil can be derived from the soil-water retention curve either by numerical differentiation (e.g., Pulido-Moncada et al., 2019) or by differentiating fitted water retention models (e.g., Dexter et al., 2008). Changes in land use influence the PSD of the soil and thereby affect a range of important soil functions such as water and nutrient availability essential for plant growth, percolation and microbial activity (Kravchenko and Guber, 2017; Rabot et al., 2018). Previous studies reveal differences in PSDs of contrasting long-term treatments (e.g., Bacq-Labreuil et al., 2018; Jensen et al., 2019). In agricultural cropping systems, land use and management vary according to the farm type specific crop rotation. In the humid temperate-region, most dairy production systems involve ley-arable rotations. Management includes establishment of grassland on arable soil and conversion of perennial grassland into arable cropping (Eriksen et al., 2015). It is in general anticipated that conversion from arable cropping to grassland has a positive effect on soil structure. Conversely, conversion of grass to arable cropping results in a loss of SOC and is hence expected to negatively affect soil structure (Poulton et al., 2018). To investigate relatively short-term changes in PSD when converting grassland to arable and vice versa a site with well-controlled conditions including well-defined land use history, and without confounding effects from differences in soil type, soil texture and climate is required. The Highfield land-use change experiment at Rothamsted Research (Highfield-LUCE) meets these unique requirements. The land use changes involved conversion to grassland from previously long-term arable or bare fallow management and conversion of long-term grassland into arable or bare fallow management. The bare fallow treatment represented an extreme reference point with regard to soil degradation. Our objective was to determine short-term soil restoration and degradation on PSD using grassland as focal point.

## 2 Materials and methods

### 2.1 The Highfield land-use change experiment

Soil was from the Highfield-LUCE at Rothamsted Research, Harpenden, UK (51°80′N, 00°36′W), a silt loam soil belonging to the Batcombe series, and classifies as an Aquic Paludalf (USDA Soil Taxonomy System) and Chromic Luvisol (WRB) (Watts and Dexter, 1997). Land uses were long-term grass (G), arable (A) and bare fallow (BF) as well as four reversion treatments which had been established as either G or A in 1949 or BF in 1959 and maintained until 2008, where grassland was introduced in arable (AG) and bare fallow soil (BFG), and grassland was converted to arable (GA) and bare fallow (GBF). The G, GA, GBF, A and AG treatments were part of a randomized block design with four field replicates, while the four BF and three BFG plots were located at one end of the experiment. For more details on soil management and the experiment, see Jensen et al. (2020). Pore characteristics for BF, A and G treatments have been reported previously in Obour et al. (2018) and Jensen et al. (2019). Jensen et al. (2020) focused on soil organic matter components and soil structural stability in the Highfield-LUCE. Soil was sampled in spring 2015 at a soil water content close to field capacity. Six 100-cm^3^ intact soil cores (61-mm diam., 34-mm height) were extracted from 0.06 to 0.10-m depth in each of four replicate plots providing 24 cores per treatment. For BFG there was three replicate plots providing 18 cores in total. The soil cores were retrieved in metal cylinders forced into the soil by means of a hammer. The cylinders were held in position by a special flange ensuring a vertical downward movement into the soil. After careful removal of the soil-filled cylinder, the end surfaces were trimmed with a knife. Subsequently, the soil cores were sealed with plastic lids, kept in sturdy containers to prevent soil disturbance during transport and stored in a 2 °C room until required for analyses.

### 2.2 Laboratory measurements

Soil texture was determined on air dry bulk soil (<2 mm) with a combined hydrometer/sieve method (Gee and Or, 2002) after removal of soil organic matter (OM) with hydrogen peroxide. The content of soil organic carbon (SOC) was measured by dry combustion using a Thermo Flash 2000 NC Soil Analyzer (Thermo Fisher Scientific). Before measuring soil water retention, the soil cores were placed on top of a tension table and saturated with water from beneath. Soil water retention was determined at -10-, -30-, -100-, -300-, and -1000-hPa matric potential using tension tables and pressure plates (Dane and Hopmans, 2002). The soil cores were oven-dried (105 °C for 24 h), and bulk density (BD) calculated. BD was corrected for mass and volume of >2-mm particles since the soil contained a significant amount of stones. The stone mass of the soil cores varied from 0.0 to 50.6 g and importantly the stone mass for e.g. G, GA and GBF was 5.1, 8.7 and 11.6 g, respectively, and thus different between the treatments. The stone mass was determined after wet sieving and drying. The stone volume was determined by means of a Lenz wide-neck bottle with conical shoulder and NS joint neck, and pycnometer head. The stone volume was calculated as the difference between the stone mass and the weight of displaced water divided by 0.998 g cm^−3^ (density of water at 20 °C). The determination of stone volume derives from Archimedes’ principle. Soil porosity was estimated from BD and particle density (PD). PD was measured for one plot from each treatment, i.e. seven analyses in total, by the pycnometer method (Flint and Flint, 2002). For the remaining plots, PD (g cm^−3^) was predicted from SOC (g kg^-1^ minerals) by a linear regression model based on the seven data points:

(1) Particle density = -0.0041*** (±0.0004) × SOC + 2.730*** (±0.008), *s* = 0.007, *R*^2^ = 0.96

where *R*^2^ is the coefficient of determination, and *s* is the standard deviation of the predicted value.

Water retention at -1.5 MPa was determined at plot level using a WP4-T Dewpoint Potentiometer (Scanlon et al., 2002) and based on <2-mm air-dry soil. Volumetric water content at each matric potential was calculated from the weight loss upon oven-drying. Pore-water suction was assumed to relate to an average pore size by the approximate relation:

(2) *d* = -3000/*h*

where *d* is the tube-equivalent pore diameter (μm) and *h* is the soil matric potential (hPa). The equation derives from the physics-based capillary rise equation of Young-Laplace.

### 2.3 Double-exponential model, calculations and statistics

The water retention data was fitted to the double-exponential model proposed by Dexter et al. (2008) (termed Dex):

(3) $$\theta =C+A_{1}e^{\left(-h/h_{1}\right)}+A_{2}e^{\left(-h/h_{2}\right)}$$

where *C* is the residual water content (m^3^ pores m^−3^ total soil volume), *A*_1_ (m^3^ pores m^−3^ total soil volume) and *A*_2_ (m^3^ pores m^−3^ total soil volume) describe the amount of textural and structural porosity, respectively, and *h*_1_ (hPa) and *h*_2_ (hPa) are the characteristic matric potentials at which the textural and structural porosity empty, respectively. The PSD predicted by the Dex model was visualized by differentiating Eq. 3 with respect to the logarithm of matric potential:

(4) $$\frac{d\theta }{d(\text{lo}{\text{g}}_{10}h)}=-\frac{A_{1}}{h_{1}}e^{\left(-h/h_{1}\right)}h\text{ln10}-\frac{A_{2}}{h_{2}}e^{\left(-h/h_{2}\right)}h\text{ln10}$$

**Fig. 1:**
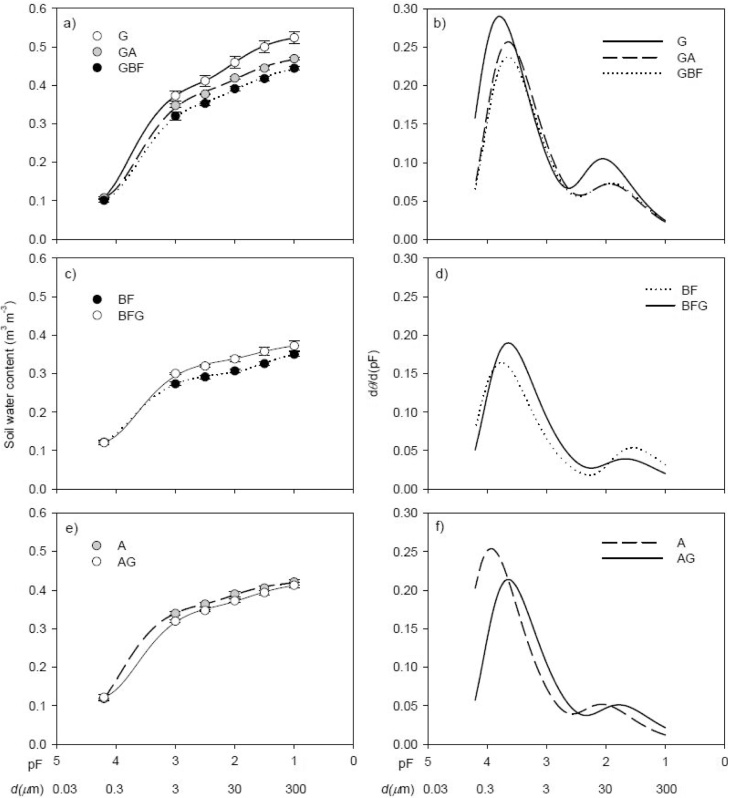
Measured volumetric water content for the comparison of G with GA and GBF (a), BF with BFG (c) and A with AG (e) and fits of the double-exponential (Dex) model as a function of matric potential (pF = log_10_(|-cm H_2_O|)). The standard error of the mean is indicated. Pore size distribution (dq/d(pF)) as a function of matric potential for the corresponding comparisons (b, d and f). Eq. 4 was used to obtain the pore size distributions. The equivalent pore diameters are indicated and were estimated by Eq. 2. For treatment abbreviations, see Table 1.

The parameters of the Dex model were obtained by nonlinear regression analysis to achieve the smallest residual sum of squares. Eq. 3 described the water retention data of the soils well (Fig. 1a, c and e), with *R*^2^ ranging from 0.997 to 1.000 and root-mean-square error (RMSE) ranging from 0.00001-0.00638 m^3^ m^−3^. We used the bi-modal Dex model rather than the widely used uni-modal model proposed by van Genuchten (1980) since the Dex model provided a better fit to the water retention data for the long-term G, A and BF treatments (Jensen et al., 2019). We evaluated the rate of change in plant available water capacity and structural void ratio. Plant available water capacity was calculated based on a soil mass equivalent to 20 cm in the G soil (abbreviated PAWC_eq_), which is analogous to how changes in SOC stocks are recommended to be assessed (Powlson et al., 2011). PAWC was determined as the difference in volumetric water content at −100 hPa and -1.5 MPa multiplied by the plough layer depth (20 cm). Subsequently, PAWC_eq_ was calculated by first calculating the mass of soil to the designated depth for all plots:

(5) *M*_soil_ = BD × 20 cm × 100

where BD is bulk density (g cm^−3^), and *M*_soil_ is the mass of soil to 20 cm depth (Mg ha^-1^). The G treatment was selected as the reference (*M*_ref_) since it had the lowest soil mass. Next, the soil mass to be subtracted from the core segment so that mass of soil is equivalent in all sampling sites was calculated:

(6) *M*_ex_ = *M*_soil_ - *M*_ref_

where *M*_ex_ is the excess mass of soil to be subtracted from the core segment. Finally, PAWC_eq_ was calculated:

(7) PAWC_eq_ = PAWC × ((*M*_soil_ – *M*_ex_) / *M*_soil_)

It is essential to report PAWC on an equivalent soil mass basis to take into account changes in BD and by that allowing a comparison of the quantity of water available to the crop for different cropping systems.

Structural void ratio (*V*_2_) was calculated as follows:

(8) *V*_2_ = *A*_2_ / (1-*P*)

where *A*_2_ (m^3^ pores m^−3^ total soil volume) is the Dex model estimate of structural porosity, and *P* is porosity (m^3^ pores m^−3^ total soil volume). *V*_2_ relates the volume of structural pores to the volume of soil solids (i.e. m^3^ pores m^-3^ volume of solids), which allows for comparisons across soils with different bulk densities as opposed to *A*_2_. This expression is analogous to the liquid ratio (Hillel, 1980) and has been used in several studies (e.g., Jensen et al., 2019; Schjønning and Lamandé, 2018).

The long-term BF, A and G treatments had reached steady-state equilibrium with respect to soil OM when the Highfield-LUCE experiment was initiated (Hirsch et al., 2017; Rothamsted Research, 2018). Hence, changes in PAWC_eq_ and *V*_2_ six years after conversion can be related to equilibrium values for these properties, whereby the rate of change in the scenarios can be revealed. The rate of change was calculated as follows:

(9) f = x / y × 100

where x and y denote the change in PAWC_eq_ and *V*_2_ after six years and at steady-state equilibrium, respectively, and if x < 0 then f = 0.

For the statistical analysis, the R-project software package Version 3.4.0 (R Foundation for Statistical Computing) was used. Treatment effects were analyzed as described in Jensen et al. (2020).

## 3 Results

Generally, contents of clay, silt and sand did not differ among soils retrieved from different treatments (Table 1) allowing treatment effects to be examined without confounding effects related to differences in soil texture.

**Table 1:** Soil characteristics and bulk density. Within rows, letters denote statistical significance at *P* < 0.05 for the comparison of G with GA and GBF, BF with BFG, and A with AG. Grass (G), grass converted to arable (GA), grass converted to bare fallow (GBF), bare fallow (BF), bare fallow converted to grass (BFG), arable (A) and arable converted to grass (AG). Soil characteristics from Jensen et al. (2020).

	G	GA	GBF	BF	BFG	A	AG
Texture^a^							
Clay <2 μm	0.261	0.255	0.254	0.270	0.244	0.264	0.266
Silt 2–20 μm	0.272^b^	0.255^a^	0.256^a^	0.249	0.267	0.263	0.253
Silt 20–63 μm	0.319	0.335	0.337	0.335	0.338	0.318	0.332
Sand 63–2000 μm	0.148	0.155	0.153	0.146	0.151	0.155	0.149
Soil organic carbon (SOC, g kg^−1^ minerals)	32.9^b^	28.2^a^	25.6^a^	9.0	13.1	17.3	18.6
SOC relative change (%)		−14%	−22%		+46 %		+8 %
Bulk density (g cm^−3^)	1.13	1.19	1.19	1.45^a^	1.54^b^	1.39	1.38

a kg kg^−1^ of mineral fraction and based on oven-dry weight.

Compared with long-term grassland (G), the SOC content decreased by 14 and 22 % (Table 1) in GA and GBF. Total porosity did not change (Table 2), whereas the PSD of the soils changed (Fig. 1b). Plant available water capacity (PAWC; water retained in 0.2−30 μm pores) decreased significantly for GA and GBF compared to G. This was mainly ascribed to 0.2−3 μm and 10−30 μm pore size classes which decreased in the order G > GA > GBF with G and GBF being significantly different (Table 2). The drop in textural porosity was reflected in a significant reduction in *A*_1_ in the GA and GBF treatments (Table 3). Similarly, the fraction of soil volume represented by 30−100 μm pores decreased significantly for GA and GBF compared to G and was reflected by a decrease in *A*_2_. The fraction of soil volume represented by pores >300 μm was significantly higher for GA and GBF than for G.

**Table 2:** Porosity in seven pore size classes and total porosity. Within rows, letters denote statistical significance at *P* < 0.05 for the comparison of G with GA and GBF, BF with BFG, and A with AG. For treatment abbreviations, see Table 1.

			G	GA	GBF	BF	BFG	A	AG
Porosity in pore size classes	>300 μm	(m^3^ m^−3^)	0.038^a^	0.075^b^	0.099^b^	0.111^b^	0.049^a^	0.054	0.067
	100−300 μm		0.024	0.025	0.026	0.024^b^	0.015^a^	0.016	0.019
	30−100 μm		0.041^b^	0.025^a^	0.026^a^	0.024	0.020	0.016	0.021
	10−30 μm		0.048^b^	0.042^ab^	0.038^a^	0.015	0.019	0.026	0.025
	3-10 μm		0.039	0.029	0.033	0.018	0.017	0.024	0.029
	0.2−3 μm		0.266^b^	0.240^ab^	0.219^a^	0.159^a^	0.181^b^	0.220	0.200
	<0.2 μm		0.101	0.107	0.101	0.116	0.119	0.119	0.119
	Total		0.561	0.544	0.543	0.460^b^	0.422^a^	0.475	0.479

**Table 3:** Fitted parameters of the double-exponential model (Dex) of the seven treatments. Within columns, letters denote statistical significance at *P* < 0.05 for the comparison of G with GA and GBF, BF with BFG, and A with AG. *d*_1_ and *d*_2_ indicate the dominating pore size of the textural and structural peak, respectively, and were estimated by Eq. 2. For treatment abbreviations, see Table 1.

	Parameters of the Dex model						
Treatment	*C*	*A*_1_	*h*_1_	*d*_1_	*A*_2_	*h*_2_	*d*_2_
	m^3^ m^−3^	m^3^ m^−3^	hPa	μm	m^3^ m^−3^	hPa	μm
G	0.080	0.343^b^	6216^b^	0.5	0.110^b^	102	29
GA	0.100	0.303^a^	4396^a^	0.7	0.075^a^	74	41
GBF	0.098	0.280^a^	4396^a^	0.7	0.078^a^	72	42
–							
BF	0.110	0.195	5768^b^	0.5	0.059^b^	35	86
BFG	0.117	0.223	4398^a^	0.7	0.047^a^	39	77
–							
A	0.068^a^	0.305^b^	8707^b^	0.3	0.050	97^b^	31
AG	0.115^b^	0.253^a^	4396^a^	0.7	0.053	53^a^	57

Introduction of grassland in bare fallow soil (BFG) led to an increase in SOC by 46 % compared to BF (close to significant, *P* = 0.053), while conversion to grassland in arable soil (AG) increased SOC marginally by 8 % compared to A (Table 1). There were no significant differences in any of the measured pore size classes nor in total porosity when A was compared to AG (Table 2); this was also partly reflected in the PSDs (Fig. 1f). For BFG, however, total porosity decreased significantly compared to BF. The PSD changed towards a significantly greater fraction of soil volume represented by 0.2−3 μm pores, and a significant reduction in 100−300 μm pores as well as >300 μm pores (Table 2). This was reflected in the PSDs (Fig. 1d) and resulted in a greater *A*_1_ for BFG than for BF (close to significant, *P* = 0.08) and a significant reduction in *A*_2_ (Table 3).

**Fig. 2:**
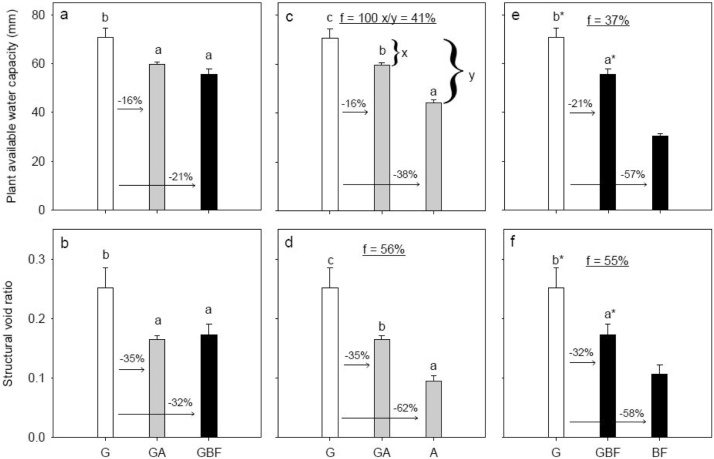
Degradation scenarios: Land use change effects on plant available water capacity calculated based on a soil mass equivalent to 20 cm in the G soil, and structural void ratio. White, gray and black bar fills represent grass, arable and bare fallow treatments, respectively, at time of sampling. Letters denote statistical significance at *P* < 0.05. An asterisk (*) indicates if BF is significantly different from GBF and G based on a pairwise *t*-test. The numbers above the arrows denote relative differences. The underlined number in the middle part of the figures denotes the rate of change, and was estimated by Eq. 9. An example of the calculation is shown in Fig. c. For treatment abbreviations, see Table 1.

**Fig. 3:**
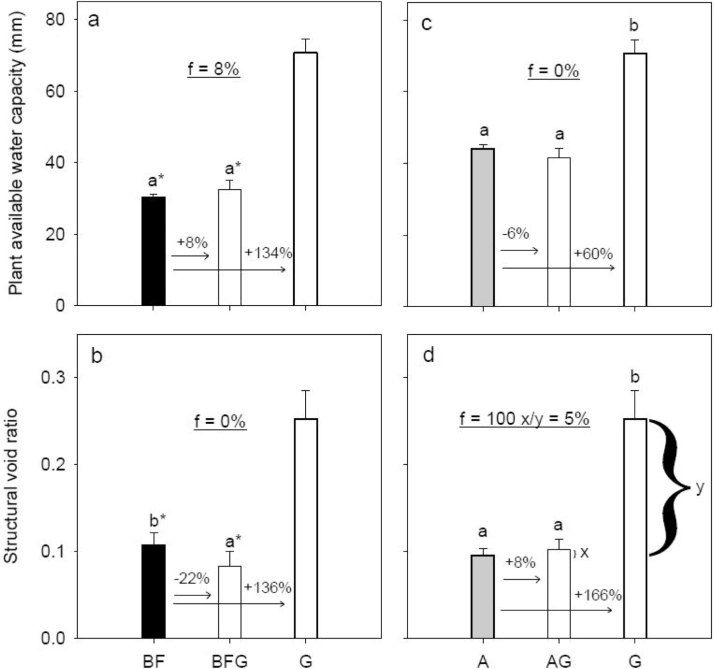
Restoration scenarios: Land use change effects on plant available water capacity calculated based on a soil mass equivalent to 20 cm in the G soil, and structural void ratio. White, gray and black bar fills represent grass, arable and bare fallow treatments, respectively, at time of sampling. Letters denote statistical significance at *P* < 0.05. An asterisk (*) indicates if G is significantly different from BF and BFG based on a pairwise *t*-test. The numbers above the arrows denote relative differences. The underlined number in the middle part of the figures denotes the rate of change, and was estimated by Eq. 9. An example of the calculation is shown in Fig. d. For treatment abbreviations, see Table 1.

For long-term grassland (G), the plant available water capacity based on equivalent soil mass (PAWC_eq_) was 71 mm water. Conversion of grassland into arable (GA) and bare fallow (GBF) soils reduced PAWC_eq_ to 60 and 56 mm water, respectively. This corresponds to relative reductions of 16 and 21 % (Fig. 2a). PAWC_eq_ for long-term bare fallow (BF) and arable (A) soils were 30 and 44 mm water, respectively. Introduction of grassland did not change these quantities significantly (Fig. 3a and c). Compared with G, the structural void ratio (*V*_2_) decreased by 35 and 32 % for GA and GBF (Fig. 2b), while *V*_2_ decreased by 22 % for GBF compared to BF and increased by 8 % for AG compared to A (Fig. 3b and d).

The changes in PAWC_eq_ for GA and GBF corresponded to approximately 40 % decrease of the range between A and BF, respectively (Fig. 2c and e). The rate of change in *V*_2_ for the same treatments corresponded to 55 % decrease (Fig. 2d and f). However, PAWC_eq_ and *V*_2_ did not change significantly for BFG compared to BF and AG compared to A (Fig. 3) apart from the reduction in *V*_2_ for BFG.

## 4 Discussion

Schjønning and Thomsen (2013) advocated expressing PAWC on a mass-equivalent basis when comparing tillage systems with significant differences in soil bulk density. In this study, PAWC_eq_ (Fig. 2, Fig. 3) represents the quantity (expressed in mm in analogy with expressions of water balances) of water available to the crop in the 0−20 cm A-horizon in the G treatment and in corresponding soil masses for the other treatments. *V*_2_ relates to the volume of soil solids whereas the corresponding *A*_2_ variable provides the traditional pore volume for a given soil volume including soil solids and voids.

Converting grassland to arable or bare fallow management decreased PAWC_eq_. This relates to a change in soil structure ascribed to reduced SOC contents and increased tillage intensity. The decrease in PAWC_eq_ for GA and GBF corresponds to a reduction of 11 and 15 mm water. Such a reduction has little impact on plant growth at this specific site because the soil type has a high PAWC_eq_ and an average annual temperature and precipitation of 10.2 °C (mean of 1992–2014) and 718 mm (mean of 1981–2010), respectively (Scott et al., 2014). However, for light-textured soils in drier regions the reduction would be more significant.

The decrease in *V*_2_ following termination of grassland may be ascribed to tillage-induced breakdown of aggregates (Jensen et al., 2020), including destruction of the enmeshment of aggregates by roots and hyphae and rearrangement of the pore system. The ∼30−90 μm pore size class has been shown to enhance microbial activity and the decomposition of OM (Kravchenko and Guber (2017) and references therein). The reduction in this specific pore size class following termination of grassland may thus negatively affect the decomposition of OM and related effects on nutrient supply and soil properties. The increase in >300 μm pores following termination of grassland may be ascribed to the increase in tillage intensity introducing very large pores.

The arrangement of pores did not show any signs of recovery six years after introducing grassland on arable soil. The introduction of grass on bare fallow soil, however, did show signs of recovery with respect to increases in textural pores (only significant for 0.2−3 μm pores), but the effect disappeared when looking at PAWC_eq_ (Fig. 3a) due to the decrease in total porosity for BFG compared to BF (Table 1). The minor changes in PAWC_eq_ caused an insignificant increase of 2 mm in BFG and an insignificant decrease of 3 mm water in AG. Based on a meta-analysis, Minasny and McBratney (2018) found that the SOC related increase in PAWC was 3−4 mm 20 cm^−1^ per 10 g kg^-1^ increase in SOC. Our study shows that the effect of SOC on PAWC_eq_ can be even smaller. The marked reduction in >100 μm pores and reduction in *V*_2_ for BFG may be related to an increase in density of the initially intensively tilled and degraded soil. The soil is in a transition phase, where a complex soil structure is developing, and the results indicate that the soil is in its initial phase with regard to the development of such a structure. The marked reduction in *V*_2_ is undesirable as root growth may be negatively affected. Further, gas exchange may be reduced potentially leading to anoxic conditions and increased potential for greenhouse gas emissions (Ball, 2013).

In essence, the results show that it was much faster to degrade both PAWC_eq_ and *V*_2_ than to restore these soil pore properties. Results on macro-aggregate stability for the same treatments, however, showed the opposite, namely that it was faster to gain than to lose stability (Jensen et al., 2020). This implies that even though the aggregates increased in stability, presumably due to the combined effect of an increase in bonding and binding agents and the lack of disturbance (Jensen et al., 2020), the soil pore network did not show signs of self-organization. The results contradicts the conceptual model for self-organization of the soil-microbe complex proposed by Young and Crawford (2004). They suggest that as substrate arrives in soil, the respiration will increase and a more open aggregated state will develop, while the structure will collapse in the absence of substrate. They indicated that the rate of change would be similar in both directions. However, our results show that the rate of change is markedly greater during degradation than restoration scenarios (Fig. 2, Fig. 3). Hence, even though the BFG and AG soils show recovery of soil microbial communities (Hirsch et al., 2017), likely an increased root density, and increased structural stability (Jensen et al., 2020) it takes a long time to develop a complex soil structure. Studies on no-till also suggest that it may take a substantial time to develop a good structure when changing from a tilled system to a system with less disturbance, and that topsoil may experience a period with increasing density (e.g., VandenBygaart et al., 1999).

We based our study on a silt loam soil with 0.26 kg clay kg^−1^ mineral fraction and a relatively evenly distributed soil mass across particle size classes (denoted a graded soil). Graded soils low in SOC may exhibit hard-setting behavior and readily compact to high densities (Jensen et al., 2019; Schjønning and Thomsen, 2013). This may explain why we see little signs of recovery when introducing grassland in degraded soil. However, some soils with >0.35 kg clay kg^−1^ mineral fraction and a clay fraction primarily consisting of 2:1 clay minerals exhibit a self-mulching behavior, and rely on natural soil processes for fragmentation (Grant and Blackmore, 1991). We do not expect that our results apply for self-mulching and highly sorted soils.

## 5 Conclusions

The Highfield-LUCE enabled us to quantify rates of change in pore size distribution six years after the land use changed for soils subjected to contrasting long-term treatments. The results showed that changing land use from long-term grassland to bare fallow or arable decreased both plant available water capacity based on identical soil quantities and structural void ratio. The conversion to grassland from long-term bare fallow or arable soil did not lead to recovery in the short-term. Thus, it was faster to lose than to develop a complex soil structure. The results underline that introducing grassland in degraded soil may induce densification in the short-term with potential negative impacts on gas exchange and root growth.

## Declaration of Competing Interest

The authors declare that they have no known competing financial interests or personal relationships that could have appeared to influence the work reported in this paper.
